# Prevalence of Depression among Chinese University Students: A Meta-Analysis

**DOI:** 10.1371/journal.pone.0153454

**Published:** 2016-04-12

**Authors:** Xian-Yang Lei, La-Mei Xiao, Ya-Nan Liu, Ya-Min Li

**Affiliations:** 1 The Second Xiangya Hospital, Central South University, Changsha, Hunan Province, China; 2 Office of the President, Central South University, Changsha, Hunan Province, China; 3 Xiangya Nursing School, Central South University, Changsha, Hunan Province, China; Universitat Wien, AUSTRIA

## Abstract

**Background:**

Depression is a major mental health issue worldwide, and university students with heavy burdens of study are at a high risk for depression. While a number of studies have been conducted regarding depression among university students in China, there is a lack of information regarding the national prevalence of depression among Chinese university students. Therefore, we performed a meta-analysis to statistically pool the prevalence of depression among Chinese university students.

**Methods:**

A systematic search of scientific databases was conducted, including Chinese Web of Knowledge, Embase, PubMed, Wanfang (a Chinese database) and Weipu (a Chinese database) to find relevant publications published between 1995 and December 2015. This was supplemented by a secondary review of the reference lists of all retrieved papers to find additional relevant citations. Studies published in either English or Chinese that provided prevalence estimates of depression in Chinese university students were considered. Prevalence estimates of each eligible study were extracted and pooled in our meta-analysis using a random-effects model.

**Results:**

A total of 39 studies conducted between 1997 and 2015 including 32,694 university students were analyzed. Our results indicate that the overall prevalence of depression among Chinese university students is 23.8% (95% CI: 19.9%–28.5%). Substantial heterogeneity in prevalence estimates was noted. Subgroup analysis revealed that the prevalence of depression among medical students is higher than among other students.

**Conclusions:**

Overall, the prevalence of depression among Chinese university students is exceedingly high. This suggests that it is imperative that more attention be given to the development of appropriate mental healthcare strategies for university students in China.

## Introduction

Depressive disorder is one of the most common mental health disorders, and the prevalence of the disease reflects the mental health of the population. Depression is a worldwide problem with a lifetime prevalence of 16.2% and a 1-year prevalence of 6.6% among the general population [1]. Depression creates a heavy burden on society as a multi-problematic disorder that leads to impairment in interpersonal, occupational, and social functioning [2–4]. University students are in a special period of life in which they grow from adolescence to adulthood and consequently make many important life decisions. During this crucial stage, university students experience enormous pressure, mostly from economic stress, academic demands, and interpersonal relationships [5]. Research indicates that mental health among university students is poorer compared to their peers around the world, with high rates of mental disorders such as depression and anxiety [6–9]. For many university students, depression can not only induce terrible feelings, such as fright, feelings of inadequacy, and anger, but it also can be connected with psychological and physical morbidities [10–11]. Previous studies have reported that depression in university students is prevalent in many countries [5, 10–14], and the prevalence appears to be increasing. Moreover, depression is associated with several severe problems in university students, notably suicidal ideation [15–16], substance abuse [17–18], and acute infectious illnesses [19]. As a result, depression among university students is a major problem, highlighting the need to explore factors that are associated with depression and to provide appropriate interventions to mitigate these factors among university students.

In China, most current university students were born after the ‘‘one-child” policy changed from an incentive-based policy to a mandatory one. The majority of undergraduates today are from single-child families [20]. Consequently, some argue that this generation of children exhibits greater recklessness and an inadequate ability to endure negative life events compared to their peers with siblings [21].

It is acknowledged that the southern region of mainland China has a better economic status and medical health care than the north region. However, whether university students in south region of mainland China are less likely to be depressed remains uncertain. Besides, the enrollment expansion in Chinese university started about in 2000 has caused inadequate employment opportunities for graduates, which may potentially increase stress and pressure in obtaining employment.

Although there is a growing number of research investigating the prevalence of depression among university students, little is known about the quantitative syntheses of overall prevalence in China. And cross-countries comparison demonstrated that Asians are less likely to report their depression than people in western countries [22], and thus lower rates of depression had been reported in Chinese university students. Primary prevention is the best and most effective strategy for depression, but it requires a careful plan of action in order to adequately improve current policies regarding depression among university students. Hence, we performed a meta-analysis that summarizes the prevalence of depression among Chinese university students in order to help develop future research priorities. Moreover, the differences of prevalence of depression on sex (male and female), region (North and South) and study period (stratified by years) were also analyzed.

## Materials and Methods

### Search strategy

A systematic literature search was conducted using Chinese Web of Knowledge, Embase, PubMed, Wanfang (a Chinese database), and Weipu (a Chinese database) to find studies that reported the prevalence of depression among Chinese university students and published between 1995 to December 2015. Articles published in either English or Chinese were considered. The following search terms were used to find relevant articles: (‘depression OR depress* OR mood OR mental OR affective) AND (‘college student’ OR ‘university student’ OR ‘undergraduate’). In addition, a secondary search was performed by reviewing the reference lists of identified papers that were potentially eligible for inclusion according to our eligibility criteria. The screening process examined titles, abstracts, and full-text of potential articles and was conducted by two authors to ensure all eligible articles were included.

### Eligibility criterion

Studies were included if they met the following eligibility criteria: 1) the study was an original epidemiological study conducted among Chinese university students; 2) the study used screening methods and/or screening tools to diagnose depression and provided a description of these screening methods; 3) the study provided a prevalence estimation (or raw data that could be used to calculate the prevalence) of depression among Chinese university students; and 4) the study was published in English or Chinese and in the past 20 years. Two authors, one of whom was designated the primary author, investigated publications independently, and any discrepancies were resolved by the primary author

### Data extraction

Relevant information from the included studies was independently extracted by two reviewers. When there was a disagreement regarding whether a study should be included for analysis, the primary author made the final decision. The following information was extracted from each eligible study: first author name, year of publication, province of China, area of China (southern or northern), number of students with depression and total sample size, percentage of male subjects, prevalence estimation, sex-specific prevalence (if available), student major (i.e., medical student or other) and grade level, and screening methods for depression.

### Statistical analysis

The estimates of prevalence extracted from studies were first transformed into a quantity using the Freeman-Tukey variant of the arcsine square root transformed proportion [23], which is suitable for both the fixed and random effects summaries [24]. This was performed because the weight of inverse variance in meta-analyses with fixed-effects models is not optimal when dealing with binary data with low proportion. Additionally, this method is useful because it allows studies that reported a prevalence of 0 to also be included in the meta-analysis since the transformed estimates of prevalence are weighted very slightly towards 50% [21].

The pooled proportion was calculated using the back-transformation of the weighted mean of the transformed proportions, using inverse arcsine variance weights for the fixed-effects model and DerSimonian-Laird weights for the random-effects model. Heterogeneity across studies was evaluated with the I^2^ statistic, which can show the variation between studies with a percentage. Heterogeneities of 25%, 50%, and 75% were considered as low-, moderate-, and high-level heterogeneity, respectively [25]. For studies with high heterogeneity, the DerSimonian and Laird random-effect model was used to calculate the summary prevalence [26]. In order to explore heterogeneity across studies, subgroup analysis was performed to determine whether there were significant differences between the subgroups. Subgroups were defined on the basis of difference in study year, sample size, area of China, student major, and sex. To enlarge the pooled sample size and examine the sex differences, a subgroup analysis of percentage of male subjects was conducted as well. Besides, sensitivity analysis was performed to test the robustness of the pooled prevalence of depression by excluding each study and rerunning the meta-analysis. Finally, publication bias was assessed using the Egger’s or Begg’s regression model [27] and a visual inspection of a funnel plot. All statistical calculations were made using Stata (version 12.0; Stata Corporation, College Station, Texas, USA).

## Results

### Study selection and characteristics

**Fig 1:**
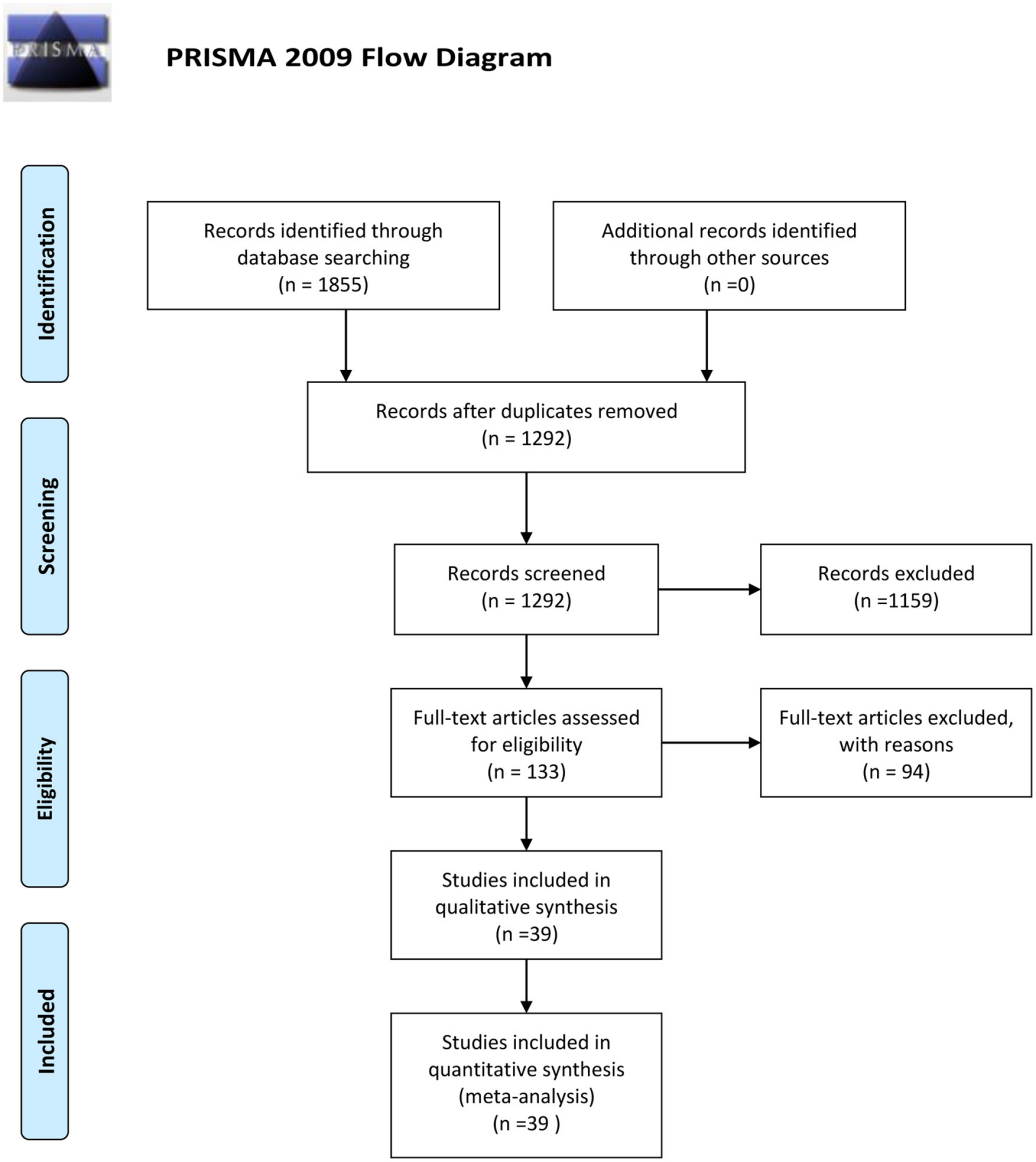
Flow diagram of the study selection process. *From*: Moher D, Liberati A, Tetzlaff J, Altman DG, The PRISMA Group (2009). *P*referred *R*eporting /terns for Systematic Reviews and *M*eta-*A*nalyses: The PRISMA Statement. PLoS Med 6(6): e1000097. doi:10.1371/journal.pmed1000097 For more information, visit www.prisma-statement.org.

A total of 1855 records were collected during the initial search, and after removal of duplicates, 1292 studies were screened by title and abstract. No new studies were retrieved during the secondary search that reviewed the bibliographies of the full-text papers collected during the initial search. In total, 39 studies, comprising 32,694 university students, were identified and included after exclusion of ineligible reports. A detailed flow chart of the search and selection process is presented in Fig 1. Table 1 presents the baseline characteristics of the 39 studies included in this analysis [28–34] (references for the 32 studies published in Chinese are presented in S1 Text). Of the 39 studies, 22 were conducted in North China and 17 were conducted in South China. In regards to type of students studied, 11 of the 39 studies targeted on medical students and 28 focused on both medical and non-medical students. The sample size of the included studies ranged from 176 to 3744. Most studies assessed the presence of depression using the Self-rating Depression Scale (SDS) [35], but several other methods were applied as well: four used the Beck Depression Inventory (BDI) [36], one used the Depression Status Inventory (DSI) [37], three used the Hamilton Depression Scale (HAMD) [38], one used Depressive Experiences Questionnaire (DEQ) [39], two used the Chinese version of the Center for Epidemiological Survey, Depression Scale (CES-D) [40], one used the Chinese version of Patient Health Questionnaire (PHQ-9) [41] and one used the revised Hopkins Symptom Checklist (SCL-90-R) [42].

**Table 1:** Characteristics of Studies on the Prevalence of Depression among Chinese College Students.

Authors and published years	Province	Area	Sample size	Age	Male percent	Prevalence			Medical student	Grade	Screening methods
			(case/total)		(%)	Total (%)	Male (%)	Female (%)			
Liu.et al.1997	Shandong	Northern	97/560	Mean(SD) 20.85 (1.81)	57.5	17.3	18.94	15.13	Yes	1–4	SDS
Du.et al.1999	Shandong	Northern	706/1597	Mean(SD) 20.56(1.40)	60.6	44.2	NA	NA	Yes	NA	BDI
Xu.et al.2002	Liaoning	Northern	31/211	Mean(range) 19.7(17–21)	38.9	14.7	17.08	13.18	Yes	1	SDS
Xu.et al.2003	Hebei	Northern	381/1750	Mean 21.8	48.3	21.8	NA	NA	No	NA	SDS
Zeng.et al.2003	Guangdong	Southern	40/302	Mean(SD) 21.08(1.12)	70.9	13.3	NA	NA	No	1–4	SDS
Zhou.et al.2003	Guangdong	Southern	71/176	Mean(SD) 21.43(0.85)	47.8	40.3	35.23	45.45	Yes	NA	SDS
Qu.et al.2005	Liaoning	Northern	114/509	Mean(SD) 20.79(1.28)	41.1	22.4	25.36	20.33	Yes	1–4	SDS
Zhang.et al.2005	Henan	Southern	615/1351	Mean(SD) 20.0(1.0)	42.7	45.5	44.73	45.74	No	1–3	SDS
Mei.et al.2006	Beijing	Southern	77/526	Mean(range) 22(20–23)	51.1	14.6	NA	NA	Yes	4	SDS
Xiao.et al.2006	Hubei	Southern	218/558	Range 16–25	44.3	39.1	44.90	34.40	No	NA	SDS
Zeng.et al.2006	Hunan	Southern	205/408	Range 17–25	55.4	50.2	50.00	50.54	Yes	1–5	SDS
Zhang.et al.2006	Guangdong	Southern	693/860	Mean(SD) 21.5(2.3)	47.4	80.6	84.6	77.0	Yes	1–4	DSI
Shi.et al.2006	Shanxi	Northern	48/658	Mean(range) 21.5(17–27)	45.0	7.3	NA	NA	No	1–5	SDS, HAMD
Wang.et al.2007	Chongqing	Southern	396/1440	Mean(SD) 20.82(2.27)	56.3	27.5	NA	NA	Yes	NA	SDS
Yang.et al.2007	Jiangsu	Southern	113/3744	Range 16–23	75.5	3.0	3.29	2.18	No	NA	SDS
Zou.et al.2007	Shandong	Northern	73/434	Mean(SD) 19.97(1.15)	45.4	16.8	NA	NA	No	NA	SDS
Zheng.et al.2008	Henan	Northern	784/1274	Mean(SD) 19.14(1.34)	25.9	61.5	NA	NA	No	1–3	SDS
Chen.et al.2009	Guangdong	Southern	381/719	NA	NA	53.0	NA	NA	No	NA	SDS
Yao.et al.2009	Hunan	Southern	157/640	Mean(SD) 20.1(1.1)	46.4	24.6	NA	NA	No	2–3	DEQ
Fu.et al.2010	Jilin	Northern	321/631	NA	51.7	50.9	NA	NA	No	NA	SDS
Niu.et al.2010	Shandong	Northern	132/609	NA	52.9	21.7	23.91	19.16	No	1	SDS
Xi.et al.2010	Hebei	Northern	84/402	NA	NA	20.9	NA	NA	No	NA	SDS
Zhong.et al.2010	Beijing	Northern	56/266	Mean(SD) 18.49(0.79)	54.7	21.1	14.49	21.85	No	NA	BDI
Chai.et al.2011	Hubei	Southern	400/1681	Range 18–22	53.5	23.8	23.11	24.58	No	1–3	SDS
Hao.et al.2011	Shanxi	Northern	214/562	Range 17–25	44.1	38.1	44.00	33.40	No	1–4	SDS
Liu.et al.2011	Shandong	Northern	54/185	Range 17–20	48.2	29.2	NA	NA	No	1	SDS
Wei.et al.2011	Fujian	Southern	151/391	Mean(SD) 20.0(2.0)	42.2	38.6	NA	NA	No	1–4	SDS
Yu.et al.2011	Shanxi	Northern	140/600	Mean(SD) 20.25(1.15)	12.5	23.3	28.00	22.67	No	1–3	SDS
Zhong.et al. 2011	Beijing	Northern	290/742	Mean(SD) 20.73(1.58)	68.6	39.1	43.02	30.47	No	1–4	HAMD
Du.et al.2013	Heilongjiang	Northern	45/650	Mean(range) 21(19–25)	66.2	6.9	NA	NA	No	NA	SDS, HAMD
Chen.et al.2013	Heilongjiang	Northern	19/477	Range 16–25	48.9	4.0	NA	NA	No	1–5	BDI
Sun.et al.2013	Hebei	Northern	510/690	Mean(SD) 21.0(2.0)	80.9	73.9	74.2	72.8	No	1–4	CES-D
Meng.et al.2013	Hubei	Southern	319/1145	Mean(SD) 21.61(1.66)	38.7	27.9	NA	NA	No	1–4	SCL-90-R
Zhang.et al.2013	Henan	Northern	84/959	Mean(SD) 21.45(1.04)	45.7	8.8	NA	NA	Yes	NA	BDI
Liu.et al.2014	Hunan	Southern	201/804	Range 17–24	45.1	25.0	26.45	23.81	No	1–3	CES-D
Feng.et al.2014	Hubei	Southern	117/1106	Mean(SD) 18.90(0.98)	57.4	10.6	NA	NA	No	1	SDS
Han.et al.2015	Liaoning	Northern	70/843	NA	29.5	8.3	NA	NA	Yes	NA	SDS
Hong.et al.2015	Beijing	Northern	398/1186	Range 15–25	35.3	33.6	34.6	34.0	No	1–4	SDS
Lu.et al.2015	Shanghai	Southern	687/1048	Mean(SD) 18.63(0.84)	66.3	65.6	65.5	65.7	No	1	Chinese version of PHQ-9

Abbreviations: BDI = Beck Depression Inventory; CES-D = Center for Epidemiological Survey, Depression Scale DEQ = Depressive Experiences Questionnaire; DSI = Depression Status Inventory; SDS = the Self-rating Depression Scale; HAMD = Hamilton Depression Scale; NA = Not available; PHQ = Patient Health Questionnaire; SCL-90-R = the revised Hopkins’ Symptom checklist

### Prevalence

**Fig 2:**
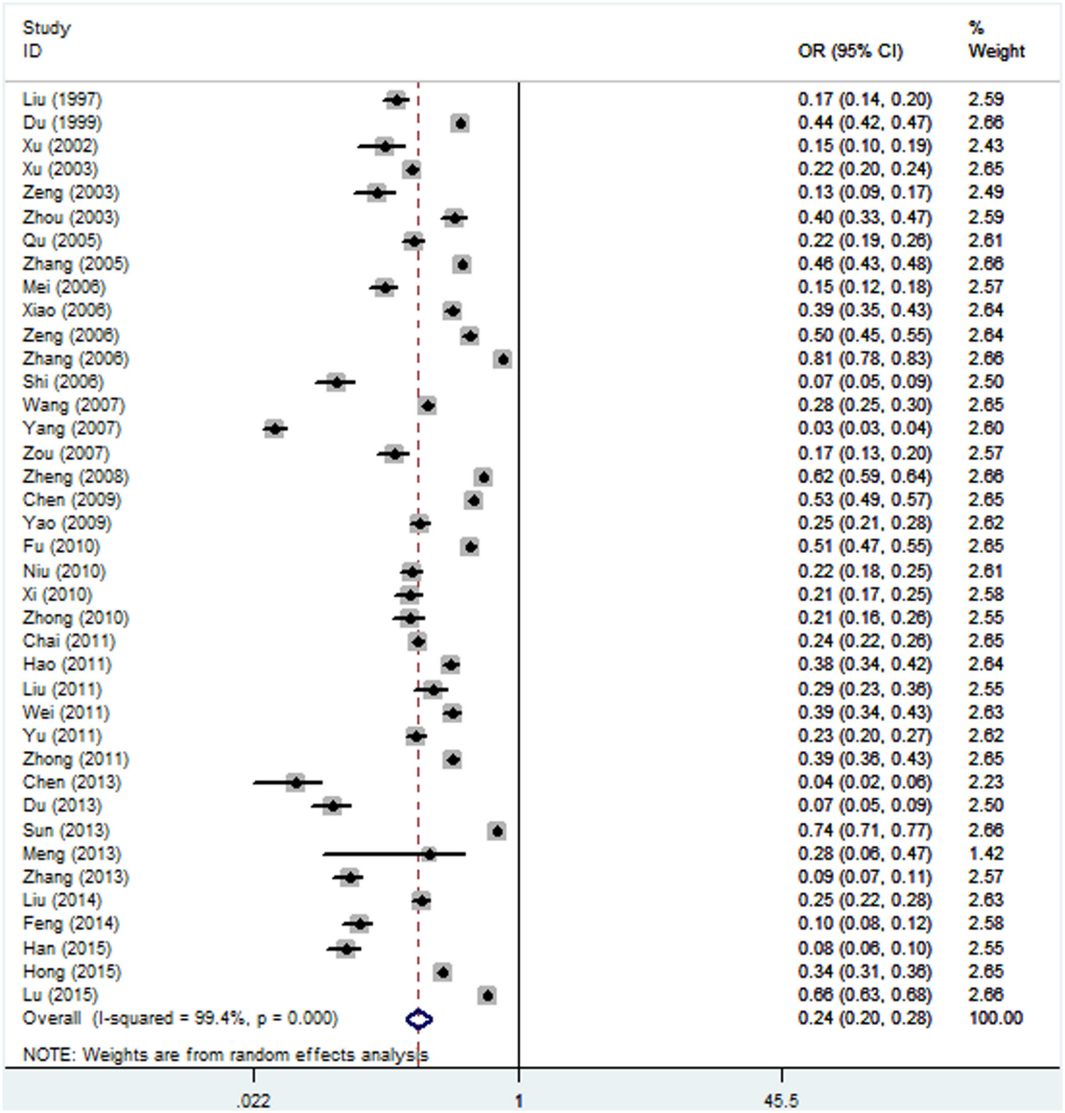
Forest plot of the prevalence of depression for the total population.

The reported prevalence of depression among the 39 individual study populations ranged from 3.0% to 80.6%, with a pooled prevalence of 23.8% (95% CI: 19.9%–28.5%, Fig 2), and there was evidence of substantial heterogeneity across the studies (I^2^ = 99.4%, P<0.001).

### Subgroup analyses

The pooled prevalence of depression among all subgroups according to study year, sex, percentage of male subjects, student major, area of China, sample size and screening method are summarized in Table 2. The pooled estimates of depression decreased over time. Between 1995 and 2000, the pooled prevalence was 27.8% (95% CI: 11.1%–69.6%), the estimate of depression was 24.0% (95% CI: 16.1%–35.8%) in studies conducted from 2001 to 2005, and 25.2% (95% CI: 18.2%–34.7%) in studies conducted from 2006 to 2010. However, the estimate was 21.9% in studies conducted during 2011–2015 (95% CI: 16.2%–29.6%), which represents a remarkable decline in depression. According to the subgroup of sex, the prevalence of depression among males (30.0%, 95% CI: 14.9%–60.2%) was a little higher than the prevalence of depression among females (27.1%, 95% CI: 13.2%–55.5%). However, the prevalence estimates in studies with samples that included fewer than 50% male subjects (24.1%, 95% CI: 18.6%–31.2%) were higher than estimates in studies with more than 50% male subjects (22.3%, 95% CI: 16.4%–30.2%). As a result, there was no significant difference between estimates of depression between males and females. The prevalence of depression among non-medical students (22.4%, 95% CI: 17.9%–28.1%) was lower than among medical students (27.5%, 95% CI: 19.8%–38.3%). Additionally, the prevalence of depression among university students from South China (27.5%, 95% CI: 20.5%–36.7%) was higher than among students from North China (21.3%, 95%CI: 16.8%–27.0%). Regarding the prevalence of depression according to sample size, the pooled prevalence for sample sizes <500, 500–1000 and >1000 were 19.8% (95% CI: 13.4%–29.3%), 25.4% (95% CI: 19.2%–33.6%), and 25.0% (95% CI: 17.9%–34.9%), respectively. Besides, no significant difference was observed between studies that have used self-reported depression instruments (24.0%, 95% CI: 19.4%–29.7%) and those that have used a clinician-rated instrument (23.7%, 95% CI: 18.0%–31.1%).

**Table 2:** Subgroup Analysis of Depression among Chinese College Students According to Different Categories.

Category	Subgroup	NO. of studies	Prevalence (95%CI) (%)	N	I^2^ (%)	P	Publication Bias Tests	
							P (Begg’s Test)	P (Egger’s Test)
	Total	39	23.8 (19.9–28.5)	32694	99.4	<0.001	0.894	0.001
Study year	1995–2000	2	27.8 (11.1–69.6)	2157	98.9	<0.001	1.000	0.001
	2001–2005	6	24.0 (16.1–35.8)	4299	98.2	<0.001	1.000	0.855
	2006–2010	15	25.2 (18.2–34.7)	13169	99.5	<0.001	0.882	0.001
	2011–2015	16	21.9 (16.2–29.6)	13069	99.3	<0.001	0.241	0.032
Sex	Male	19	30.0 (14.9–60.2)	9430	99.9	<0.001	0.033	0.001
	Female	19	27.1 (13.2–55.5)	7559	99.9	<0.001	0.220	0.001
Male percent	<50%	21	24.1 (18.6–31.2)	15573	99.3	<0.001	0.833	0.047
	≥50%	16	22.3 (16.4–30.2)	16000	99.5	<0.001	0.652	0.001
Medical students	Yes	11	27.5 (19.8–38.3)	9054	99.4	<0.001	0.640	0.169
	No	28	22.4 (17.9–28.1)	23660	99.3	<0.001	0.707	0.001
Area	Northern	22	21.3 (16.8–27.0)	15795	99.1	<0.001	0.352	0.006
	Southern	17	27.5 (20.5–36.7)	16899	99.5	<0.001	0.117	0.001
Sample size	<500	10	19.8 (13.4–29.3)	3425	97.7	<0.001	0.025	0.001
	500–1000	18	25.4(19.2–33.6)	11947	99.4	<0.001	0.023	0.002
	>1000	11	25.0 (17.9–34.9)	17322	99.5	<0.001	0.696	0.001
Screening method	Self-report	26	24.0 (19.4–29.7)	22158	99.1	<0.001	0.415	0.001
	Clinical instrument	13	23.7 (18.0–31.1)	10536	99.4	<0.001	0.300	0.090

### Sensitivity analysis

**Fig 3:**
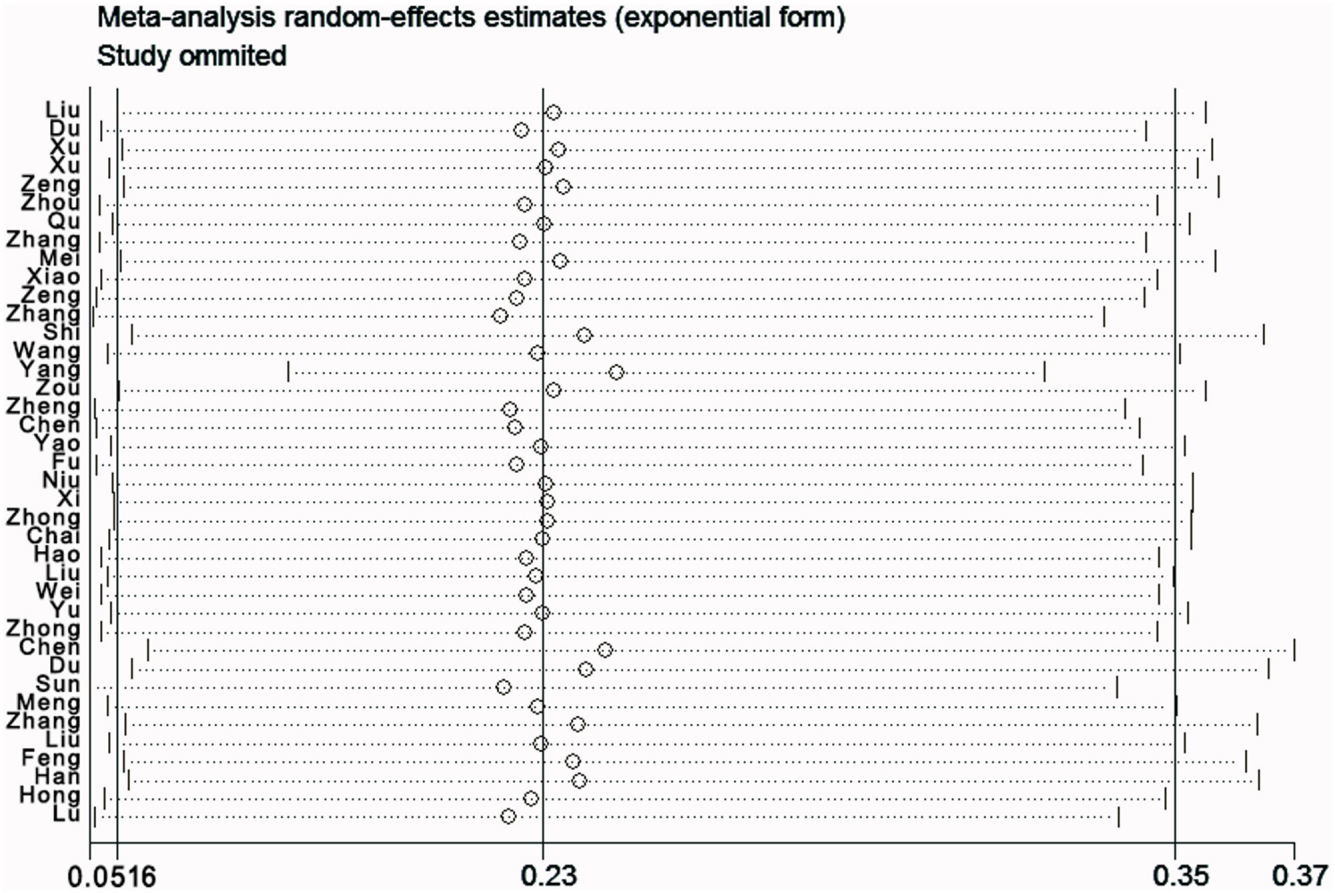
Plot of sensitivity analysis by excluding one study each time and the pooling estimate for the rest of the studies.

To examine the stability of the pooled prevalence of depression, each study was sequentially excluded. And the results of sensitivity analysis demonstrated that none of the exclusions of any specific study evidently altered our results, which revealed that the prevalence of depression among Chinese university students is exceedingly high (Fig 3).

### Publication bias

**Fig 4:**
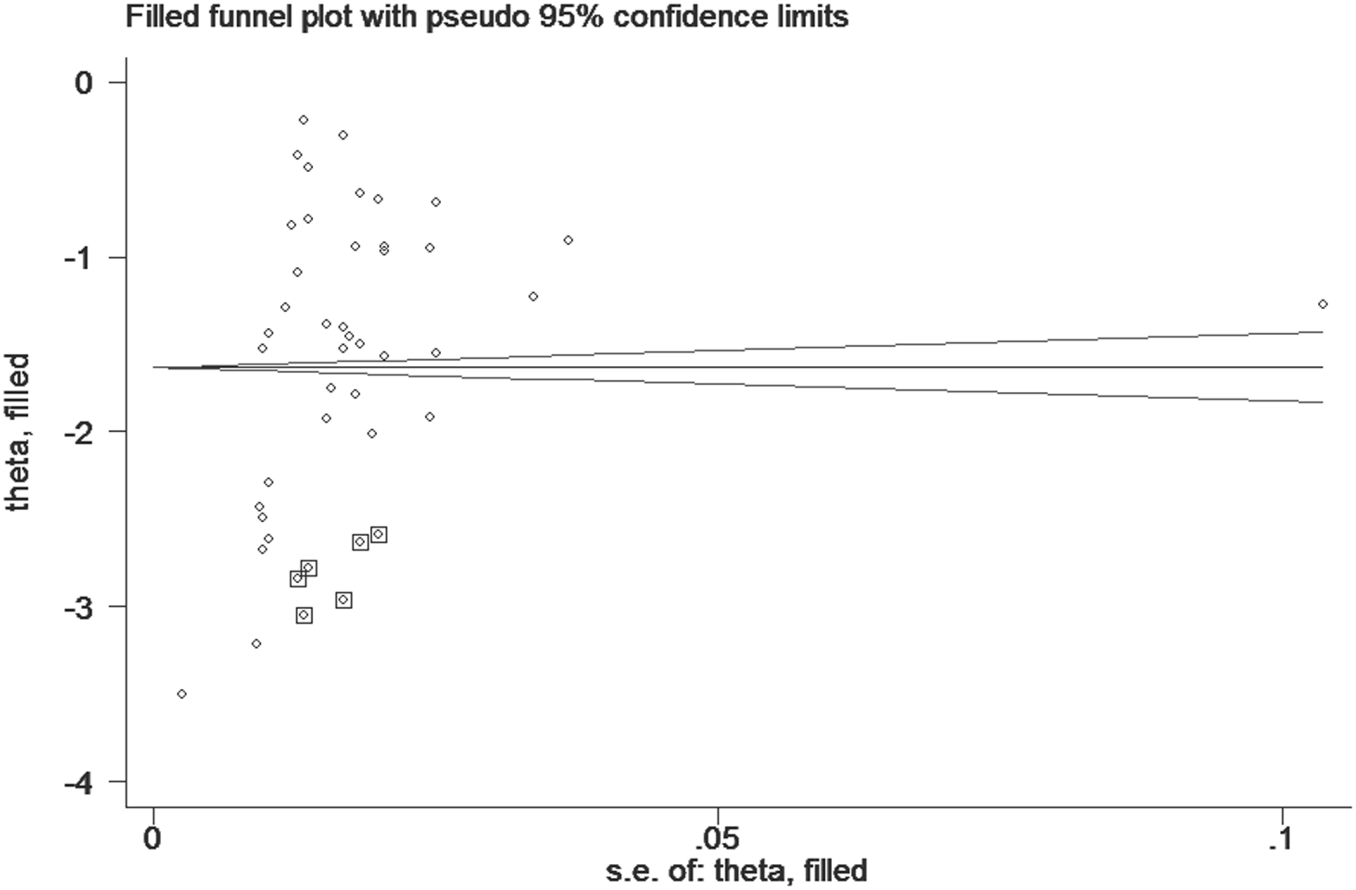
Filled funnel plot of the included studies (log odds ratio, horizontal axis; standard error, vertical axis).

The publication bias of each study was first assessed using Begg’s funnel plot. Strong evidence of asymmetry was revealed by the shape of the funnel plot, suggesting the presence of publication bias. Then, the trim and fill method was applied to further quantitatively assess publication bias by filling the funnel plot using the random-effects model, estimating six potential studies were missing (Fig 4). The recalculated overall prevalence of depression according to the trim and fill method was 19.5% (95% CI: 13.8%–27.7%). Overall, it is likely that publication bias exists among the included studies according to the above statistical results.

## Discussion

This meta-analysis of 39 epidemiological studies is the first systematic report summarizing the prevalence estimations of depression in Chinese university students. This comprehensive meta-analysis of observational studies conducted in China during the last 20 years included more than 30,000 university students. This meta-analysis is likely to provide a trustworthy estimate of the prevalence of depression among Chinese university students. The results of this meta-analysis indicate that the prevalence of depression among Chinese university students is 23.8% (95% CI: 19.9%–28.5%), which is close to earlier reported estimates and falls within the range of 10%–85% that was reported in another systematic review of depression in university students worldwide, but the prevalence is relatively lower than estimates of the global prevalence of depression (weighted mean prevalence: 30.6%) [14].

Negative life events may play a significant role in the development of depression. Compared to non-depressed university students, students with depressive symptoms often reported experiencing more negative events [28]. More so, university students with depressive symptoms also stated that negative life events had a great impact on their lives, suggesting that they not only experienced a high frequency of negative life events but also felt a great degree of stress resulting from these negative life events [43]. Findings also suggested that negative life events, such as failure in love, separation of parents and financial deficits, were direct source of stimulation inducing depression. University students with more negative life experiences are at increased risk for developing depression [44].

Some studies have found higher levels of depression among female students than male students [12, 45]. However, we found no significant difference in the prevalence of depression between female and male sexes in the current study. Similarly, previous studies have observed no differences in depressive symptoms between male and female students [6, 10]. This could be explained by the fact that female students in Chinese universities enjoy equal political rights, experience the same pressures as male students, and have same job opportunities as their male peers [30].

Our study revealed that the prevalence of depression among medical students was higher compared to other students. Several theories explaining this trend have been presented in many studies. Some have attributed this result to the fact that medical students go through continuous examinations throughout their academic study. Studying medicine is relatively more competitive compared to other majors of study, and several unique academic stressors have been reported in various previous studies [12]. Students in other disciplines undergo fewer examinations and easier courses of study compared to medical students. Others have stated that medical students are more likely to be critical of themselves. Anecdotal evidence suggests that medical students tend to be more socially isolated than other students. Besides, medical education may inevitably have negative effects on mental health and increase the risk of depression among medical students [14, 46]. The cumulative impact of these various factors may explain why medical students experience higher rates of depression compared to students in other disciplines.

Subgroup analysis also revealed that the prevalence of depression in southern areas of China was higher compared to than in northern areas of the country. Similar results have not been found in previous studies, as nationwide investigation on this topic is scarce. This variance could be explained by differences in education, family income, medical insurance, and other social-culture factors between southern and northern part of China. However, further research is required to confirm the difference in the prevalence of depression between southern and northern areas of China and to evaluate the factors that might lead to such differences. This may be useful in the development of primary prevention strategies.

Even though this meta-analysis included 39 studies and encompassed a larger sample size than any individual study, some limitations deserve to be acknowledged and discussed. First, substantial heterogeneity was observed in both the overall analysis and subgroups analyses. Some of the selected studies had limited sample sizes that produced imprecise estimates. And part of the heterogeneity across various studies could be explained by the diverse sample sizes and sex ratios. In addition, the differences in screening methods among the various studies may have contributed to the observed heterogeneity. Some factors that may have contributed to the heterogeneity must be acknowledged.

A second possible limitation of this study is related to publication bias. Although the results from Egger’s test and Begg’s test were inconsistent, there was evidence of publication bias, mostly from language bias resulted from unbalanced number of studies published in different languages (32 in Chinese and 7 in English) and the inflated estimates resulted from limited methodological design in some studies, The included studies had noted methodological flaws, especially related to selection and recruitment of university students with depression. The samples of students participating in the investigations may not be representative of other student samples in China. This may have led to subjects being included in studies who differed in important ways from those who were excluded or ineligible for statistical analyses. As a result, the estimates of prevalence may have been influenced in unpredictable ways [21].

In conclusion, the prevalence of depression among Chinese university students is high, mostly resulted from the expanded social interactions and changing situations in residence and finance. Future studies in this field should be more focused on the characteristics of the participants to find key factors that contribute to the prevalence. In consideration of the new policies that aim to expand enrollment at universities in China are being implemented, we hope this meta-analysis that summarizes research over the past 20 years will serve as an alarm bell to the university administration. The results of our study should stimulate not only more research on university students as a distinct group, but also encourage families, universities, and society as a whole to develop and implement strategies to help the young to overcome their difficulties and lead a healthier life.

## Supporting Information

S1 Table PRISMA 2009 Checklist. (DOC)

S1 Text References from 32 Chinese studies that were included in the meta-analysis. (DOCX)
