# Supplementary material for: Prevalence of Depression among Chinese University Students: A Meta-Analysis
Source: PLoS One. 2016 Apr 12;11(4):e0153454. doi: 10.1371/journal.pone.0153454 (PMC4829172; doi:10.1371/journal.pone.0153454)
Supplement: S1 Text — (DOCX) [file pone.0153454.s002.docx]

Supplement 1. References from 31 Chinese studies that were included in the meta-analysis

1. Liu CX, Tang MQ, Hu L, Wang AZ, Li CQ. Association between anxiety, depression and sleeping quality in college students. Chinese Mental Health Journal. 1997; 11: 25-27.

2. Du ZY, Wang KQ. An epidemiological study on depression in 1597 undergraduates. Chinese Journal of Behavioral Medical Science. 1999; 8(3): 172-173.

3. Xu LM, Guan WP, Wei XQ, Li WY, Sun X. An investigation of anxiety and depression in lower grade students in medical school. Health Psychology Journal. 2002; 10: 24-25.

4. Xu Y, Zhu XX, Zhao JB, Liu LY. An investigation of mental health in 1750 undergraduates. Chinese Journal of Primary Medicine and Pharmacy. 2003; 10: 1073.

5. Zeng LN. Study on depressed symptom and its influencing factors among sport professional college students. Chinese Journal of School Doctor. 2003; 17: 393-394.

6. Zhou R, Yang CH, Pan JY. An investigation of depression and relevant factors in key medical school in Guangzhou. Nervous Diseases and Mental Hygiene. 2003; 3: 367-368.

7. Cui DC, Pan XD, Li HY, Mou J, Zhou XS. Survey on psychological and social factors of depression in medical college students. Chinese Journal of Public Health. 2005; 21: 392-393.

8. Zhang QJ, Incidence rate of depression and related factors analysis in undergraduates of different crowd from normal university. Chinese Journal of Clinical Rehabilitation. 2005; 9: 92-93.

9. Mei L, Zhang FY, Liang Y, Zhang BY, Wang XQ, Tan HR. Analysis of the mental health status between medical and non-medical students. China Medical Equipment. 2006; 3: 46-48.

10. Xiao WZ, Zhou H, Xia Y, Yuan J, Yan Q. Depression status and its risk factors among the college students. Chinese Journal of Behavioral Medical Science. 2006; 15: 647-649.

11. Zeng Q, Li L, Tang SY, Long LL, Xue Jh, Long B et al. Analysis on Influential Factors and Prevention Way of Depression Among College Students in a Certain University. Practical Prevention Medicine. 2006; 13: 1412-1413.

12. Zhang ZW, Wu CP, Liu GN, Fan CX, Wang HS. Depression and the influencing factors of medical college students in Guangzhou. Chinese Journal of School Health. 2006; 27: 51-52.

13. Shi JF. Analysis on the prevalence of depression and influential factors of physique among college students. Chinese Journal of Management in Chinese Medicine. 2006; 14: 21-22.

14. Wang T, Wang NW, Hu HQ, Feng ZZ, Liu YB. Relationship of depression, automatic thoughts and personality in medical college students. ACTA ACADEMIAE MEDICINAE MILITARIS TERTIAE. 2007; 29: 442-444.

15. Yang XH. An epidemiological investigation of depression among 3744 freshmen. China Journal of Health Psychology. 2007; 5: 499-500.

16. Zhou FR, Wang Y, Zhang JX, Liu LF, Qiu HM, Liu JT. A Study of Psychological Education on Anxiety and Depression of University Students. Chinese Journal of Clinical Psychology. 2007; 15: 557-558.

17. Zeng TF, Zhou QY. Study on mental health status of college student and its influential factors. Modern Preventive Medicine. 2008; 35: 4825-4827.

18. Chen JS, Peng DY, Liang YB, Tan YS, Wei ZY, Dong L. Investigation and analysis on smiling depression of college students in Guangzhou city. Chinese Journal of General Practice. 2009; 7: 191-192.

19. Fu GF. The Relationship between Undergraduate Students' Social Supports and Depression. Medicine and Society. 2010; 23: 83-90.

20. Niu RH, Jiang GL, Fang ZY. Status of coping style, anxiety and depression of college freshmen and their relationship. Chinese Journal of Health Education. 2010; 26: 548-549.

21. Xi MJ. Analysis on depression status and causes in college students in Xingtai University. Journal of Xingtai University. 2010; 25: 12-13.

22. Chai XR. Investigation and analysis on depression among students in Jianghan University. Journal of Yangtze University. 2011; 8: 198-200.

23. Hao LL, Hao WP. Depressive emotion and correlation factors in college students. Journal of Clinical Psychosomatic Diseases. 2011; 17: 342-344.

24. Liu BF, Lin J, Zhang ZH, Xu B, Duan XL, Liu FH et al. Study about related factors of depression and anxiety in university freshmen in Shandong province. Journal of Psychiatry. 2011; 24: 100-101.

25. Wei YX, Yan YW, Lin RM, Liu L. Correlation between depression and sleep quality among college students in Fuzhou City. Chinese Journal of School Doctor. 2011; 25: 172-175.

26. Yu XF. Depressive symptoms and related factors analysis in English major college students. Chinese Journal of Disease Control and Prevention. 2011; 15: 145-147.

27. Zhong SF, Tu WJ, Shi XD, Zhang WJ. Study on related factors of depression in public security college students. China Journal of Health Psychology. 2011; 9: 1382-1384.

28. Du LJ. Features of depression in traditional Chinese medicine and associated influential factors in college students. World Health Digest. 2013; 10: 366.

29. Sun LJ. Analysis of distribution of depression group in college students. Journal of Hubei University of Economics. 2013; 10: 153-154.

30. Liu AM. Analysis on depression and influential factors in students of Hunan University of Science and Engineering. Journal of Hygiene Research. 2014; 43: 508-511.

31. Han YS, Lai R, Zhao DS, Liu C, Xu C, Yu Q. Reason analysis and prevention of depression among students in a medical college. Journal of Shenyang Medical College. 2015; 17: 56-60.

32. He H, Luo Y. Analysis on depression status and influence factors of university students in Beijing. Modern Preventive Medicine. 2015; 42: 1261-1266.
